# Polymorphisms of the Proinflammatory Cytokine Genes Modulate the Response to NSAIDs but Not to Triptans in Migraine Attacks

**DOI:** 10.3390/ijms24010657

**Published:** 2022-12-30

**Authors:** Elisa Rubino, Andrea Marcinnò, Alberto Grassini, Elisa Maria Piella, Fabio Ferrandes, Fausto Roveta, Silvia Boschi, Aurora Cermelli, Salvatore Gallone, Lidia Savi, Innocenzo Rainero

**Affiliations:** 1 Department of Neurosciences “Rita Levi Montalcini”, University of Torino, 10126 Torino, Italy; 2 Department of Neuroscience and Mental Health, Città della Salute e della Scienza di Torino, 10126 Torino, Italy

**Keywords:** migraine, polymorphism, cytokines, TNF-α, NSAIDs, triptans

## Abstract

Migraine is a common neurovascular disorder characterized by recurrent episodes of headache and associated neurological symptoms. At present, a significant portion of patients do not obtain a satisfactory response to acute pain-relieving therapies, including NSAIDs and triptans. In this context, pharmacogenetics plays a key role in the understanding of such a diverse response. In order to investigate whether functional polymorphisms in proinflammatory cytokine genes (*IL-1α*, *IL-1β*, *IL-1RN*; *IL-6* and *TNF-α*) may influence the response to acute treatment, 313 consecutive patients with episodic migraine without aura were enrolled. Pain relief by administration of NSAIDs or triptans for three consecutive migraine attacks was evaluated. We found a significant association between A allele of the *TNF-α* promoter (−308 A/G) and a lack of efficacy after NSAID administration (*p* < 0.01, OR 2.51, 95% CI: 1.33 < OR < 4.75 compared to the G allele). Remaining polymorphisms had no significant effect on pain relief. Our study showed that a functional polymorphism in the *TNF-α* gene significantly modulates the clinical response to NSAID administration in acute attacks. Patients with higher production of the active cytokine during stress showed a significantly lower anti-migraine effect. Our results further support a role for TNF-α in the pathophysiological mechanisms of migraine attack.

## 1. Introduction

Migraine is a chronic brain condition characterized by the recurrence of throbbing unilateral headache attacks associated with the presence of photo- and phonophobia, nausea and/or vomiting. Headache may or may not be preceded by transient focal neurological symptoms defined as migrainous aura [1]. Migraine can be also classified based on the frequency of monthly headache days as episodic (EM) or chronic migraine (CM). Migraine has been recognized as one of the leading causes of disability worldwide according to the Global Burden of Disease study [2]. Because of the significant social and economic impact, numerous studies have been conducted in order to shed light upon the pathophysiological aspects underlying the condition.

At present, frontline therapies in migraine’s acute treatment include NSAIDs and triptans [3]. Several studies showed that a significant portion of treated patients do not obtain consistent pain relief [4], and headache patients react differently to given drugs, leading to a wide variety of treatment responses. It is estimated that 25 to 50% of migraine patients adequately respond to acute therapy with NSAIDs. Pharmacogenetics bears great promise in the optimization of individual-specific therapy and in providing new targets for drug development [5]. However, the role of pharmacogenetics for therapy success with compounds used for the treatment of headache disorders is not as largely investigated, and pharmacogenetic information is hitherto practically not used to predict efficacy or adverse effects in clinical practice in this field [6,7].

Although triptans represented an important step forward in migraine management, the clinical response, defined as 2 and 24 h pain-free or pain relief plus the presence or not of recurrence, is poor in approximately one third of treated patients [8], and a wide range of inter-individual variability in both efficacy and tolerability has been described. This variability may be at least partially explained by pharmacogenomic reasons, as a few studies, analyzing polymorphisms in genes involved in the neurotransmitter systems and in the metabolism of triptans, have suggested [7,9,10]. Several other studies have dealt with the pharmacogenomics of NSAIDs, though focusing on NSAIDs’ metabolism and the polymorphisms influencing adverse reaction rates [11,12].

NSAIDs represent by far the most commonly used medications for the acute treatment of migraine. Contrariwise, triptan use in migraine attacks is still scarce [13]. Previous studies showed that around 95% of migraine sufferers use acute treatment, although only a minority of patients use specific agents, and the overall level of satisfaction with acute treatment is low [1,2].

Neurogenic inflammation is presumed to play an important role in the pathogenesis of migraine attacks, involving the release of various vasoactive neuropeptides such as calcitonin gene-related peptide (CGRP), substance P (SP), vasoactive intestinal peptide (VIP) and pituitary adenylate cyclase-activating polypeptide (PACAP). Then, a cascade of events occurs, characterized by vasodilatation, plasma protein extravasation and the release of proinflammatory mediators [14]. The role of cytokines in migraine is evidenced by the effectiveness of NSAIDs in symptomatic therapy, by the presence of ictal and interictal increased plasma levels of TNF-α and IL-6 [15] and increased levels of TNF-α in CSF [16].

The aim of this study was to investigate whether functional polymorphisms in the IL-1 family (*IL-1α*, *IL-1β* and *IL-1RN*), *IL-6* and *TNF-α* genes may influence the response to oral NSAID administration or triptans during migraine attacks.

## 2. Results

### 2.1. Clinical Characteristics and Pharmacological Response

We enrolled in the study 313 consecutive patients with episodic migraine without aura (217 females, 96 males; mean age ± SD: 38.36 ± 11.76). The timeline of recruitment and conduction of the study is reported in Supplementary Figure S1. The mean number of monthly migraine days (MMD) was 4.89 ± 3.14 and the monthly mean number of acute treatment medications consumed by the patient was 4.12 ± 2.13. Clinical details are displayed in Table 1 and Supplementary Tables S1 and S2. Migraineurs underwent clinical interviews, neurological examination and psychometric tests (data are shown in Supplementary Materials, Table S3). In the comparisons of the clinical characteristics between females and males, we observed a significant difference in headache duration (*p* = 0.009) and in the presence of nausea and vomiting (*p* = 0.007 and *p* = 0.004, respectively).

According to NSAID pharmacological tests, the 313 migraineurs were divided into three groups, according to their clinical response to oral NSAIDs: 65 patients (20.8%) were classified as “NSAID non-responders”, 125 (39.9%) as “NSAID partial responders”, and 123 (39.3%) as “NSAID responders”. In the comparisons of the responses between females and males, we observed a difference in NSAID response (*p* = 0.02).

Twenty-three patients did not receive triptan medication for acute attacks, due to the presence of concomitant vascular diseases. Thence, 290 migraineurs (207 females, 83 males) were also divided into three different groups according to their clinical response to oral triptan administration. Eighty patients (27.6%) were classified as “triptan non-responders”, 22 (15.2%) as “triptan partial responders”, and 166 (57.2%) as “triptan responders”. When we compared females to males, we observed a difference in the distribution of the triptan response according to gender (*p* = 0.003).

Overall, 34.5% of patients consuming NSAIDs have required rescue medications, and 21.4% of patients assuming triptans have required rescue therapy. Rescue medications were drugs previously used by patients for their migraine attacks (e.g., analgesics such as acetaminophen and tramadol, other NSAIDs such as nimesulid, dexketoprofen, ketoprofen, combined therapy such as indomethacin/caffeine/prochlorperazine, paracetamol/propyphenazone/caffeine, metoclopramide and domperidone for nausea).

### 2.2. NSAID Response and Genotyping

Table 2 shows genotypic (GF) and allelic (AF) frequencies of the *TNF-α* promoter −308 A/G polymorphism in patients with episodic migraine according to the NSAID response. A significant difference was found in the distribution of genotypic and allelic frequencies and the clinical response to oral NSAIDs.

Interestingly, episodic migraine patients carrying the A allele of the *TNF-α* promoter −308 A/G polymorphism showed a significant association with a lack of efficacy after NSAID administration in migraine attacks compared to the G allele (chi-square: 8.527, *p* = 0.003, OR 2.51, 95% CI: 1.33 < OR < 4.75, NSAID non-responders vs. NSAID partial responders and responders; OR 2.31, 95% CI: 1.12 < OR < 4.81, NSAID non-responders vs. NSAID responders; OR 2.74, 95% CI: 1.29 < OR < 5.84, NSAID non-responders vs. NSAID partial responders). 

Conversely, the allelic and genotypic distributions of functional polymorphisms in *IL-1α* (−889 C > T), *IL-1β* (−511 C > T), *IL-1β* (+3953 C > T), *IL-1RN* (VNTR), *IL-6* (−174 G > C) were not significantly different between the NSAID responders, NSAID partial responders and NSAID non-responders (*p* > 0.05 in all comparisons) (Table 2). Genotypic frequencies of polymorphisms of *IL-1RN* (VNTR), in non-responders, responders and partial responders to NSAIDs are displayed in Supplementary Table S4. No differences were found between the genotypic distribution and the gender response to NSAIDs.

### 2.3. NSAID Response and Clinical Characteristics

The response to NSAIDs was not related to the patients’ educational level, employment status, age and age at onset of the disease. The response to NSAIDs was not clearly related to the duration of the disease (*p* = 0.07) or to the severity of the attacks (*p* = 0.08). No difference was found in the psychometric tests for depression and anxiety among the three groups of examined patients.

### 2.4. Triptan Response and Genotyping

Allelic and genotypic frequencies of *TNF-α* (−308 G > A), *IL-1α* (−889 C > T), *IL-1β* (−511 C > T), *IL-1β* (+3953 C > T), *IL-1RN* (VNTR), and *IL-6* (−174 G > C) were not significantly different between the triptan responders, triptan partial responders and triptan non-responders (*p* > 0.05). The observed GF and AF of the polymorphisms are listed in Table 3. Genotypic frequencies of polymorphisms of *IL-1RN* (VNTR), in non-responders, responders and partial responders to triptans are displayed in Supplementary Table S4. No differences were found between the genotypic distribution and the gender response to triptans.

### 2.5. Triptan Response and Clinical Characteristics

The response to triptans was not related to the patients’ educational level, employment status, age and age at onset of the disease. The response to triptans was influenced by the presence of a pulsating characteristic of pain and photophobia, which predicted a poor response (*p* < 0.01). No difference was found in the psychometric tests for depression and anxiety and the pharmacological responses.

## 3. Discussion

To our knowledge, this is the first study that has investigated whether functional polymorphisms in proinflammatory cytokine genes may influence the response to acute treatment in migraineurs. We showed that a functional polymorphism in the *TNF-α* gene significantly modulates the clinical response to NSAID administration in acute migraine attacks. Patients with episodic migraine carrying the A allele of the *TNF-α* promoter −308 A/G polymorphism showed a significant association with a lack of efficacy after NSAID administration in migraine attacks. Our results further support a role for the proinflammatory cytokine TNF-α in the pathophysiological mechanisms of migraine attack. This is the first study using a pharmacogenetic approach for NSAID response in migraine attacks, and additional studies are needed to confirm our data. Conversely, we did not find an influence of cytokine polymorphisms in the response to acute treatment with triptans.

It is not well known whether common genetic variants can explain the difference in response to migraine acute therapy. Gene polymorphisms can influence the functionality, distribution or amount of the encoded protein, affecting the corresponding biological processes. It has been shown in earlier studies that the *TNF-α* −308 A allele is associated with increased TNF-α production as compared to the G allele [17]. In our patients with episodic migraine without aura, it could be hypothesized that migraineurs carrying the A allele showed a significantly lower anti-migraine effect of NSAIDs due to higher production of the active cytokine during stress. It could be of interest to evaluate whether these non-responding patients may benefit from a higher dosage of NSAID therapy.

The pain-free response at 2 h after NSAID administration in our patients was 39.3%. This percentage of response was higher with respect to the previous ones described in the literature [18,19]; however, it is known that when migraine attacks are treated in the early phase of headache, the 2 h pain-free responder rate increases considerably. In an animal model of migraine, the expression of COX-2, MMP9 and TNF-α increased after an NO donor infusion, with expression being more evident in the meningeal blood vessels. Our group previously reported that the –308 G allele of *TNF-α* was associated with migraine [20]. In further studies, conflicting data have been reported on the genetic association between *TNF-α* –308 G > A and migraine, whereas a recent meta-analysis showed that the A allele was a risk factor for migraine [21,22,23].

Recently, the elevation of TNF-α in the CSF of patients with new daily persistent headache and with chronic migraine was found [24]. Interestingly, the concentration of the protein in serum was normal, suggesting a role for TNF-α in the maintenance of CNS inflammation. The authors suggest that the persistent elevation of TNF-α in the CSF may be one of the causes of treatment-refractory chronic headache. A further understanding of the role of TNF-α may have therapeutic implications for future migraine treatment.

In our population, we did not find an influence of cytokine polymorphisms in the response to the acute treatment of migraine attacks after the administration of triptans. In the literature, a previous study evaluating different *MAO-A* polymorphisms (leading to MAO-A-high or MAO-A-low activity) in 124 female migraine patients did not show any influence on the efficacy or the risk of abuse of triptans [25]. At the same time, patients with the −163 A allele polymorphism in *CYP1A2* have been shown to have higher enzyme activity and a higher risk of abuse compared with patients carrying the −163 C allele [25]. Ishii et al. showed that a non-synonymous polymorphism in dopamine receptor 2 is a negative predictive factor in the response to triptans in migraineurs [9]. Furthermore, the rs2651899 polymorphism in the *PRDM16* gene encoding a zinc finger transcription factor appears to be associated with better therapeutic efficacy of triptans in migraine, as shown in a Danish study including 1,806 patients, of which 1,386 showed an effect of triptans and 376 showed no effect [7]. Furthermore, a recent study showed no evidence that variants F124C and A-161T of the 5-HT receptor are involved in the clinical response to sumatriptan. Thence, different pathogenetic pathways seem to influence the response to triptans.

In this study, we also showed that the pulsating characteristics of pain and the presence of photophobia were each associated with a poor response to triptans. These findings confirm eletriptan and sumatriptan studies, in which the presence of photophobia in an attack predicted a scarce response [26].

There are some limitations of the study that suggest caution in the interpretation of the results. This is the first study demonstrating a lack of efficacy after NSAID administration in migraine attacks in migraineurs carrying a functional polymorphism in the *TNF-α* gene, and, based on samples limited to one population, our findings and subsequent conclusions must be viewed cautiously, and further studies with a larger sample size are needed in order to confirm our data. Second, in this pilot study, the analysis was limited to patients with episodic migraine. Migraine represents a dynamic process of dysmodulation and sensitization, and our findings could change according to the chronification of the disease.

## 4. Materials and Methods

### 4.1. Enrolled Patients

A group of 340 consecutive patients with episodic migraine without aura were recruited at the Headache Center—Neurology I, Department of Neuroscience “Rita Levi Montalcini”, University of Torino, Italy. Diagnosis was made according to the International Classification of Headache Disorders, 3rd edition (IHCD-3 criteria [1]).

In the first visit, migraineurs were prescribed NSAIDs or triptans depending on their previous clinical experience and on the clinician’s indication. If one or more NSAIDs or triptans previously were anamnestically not efficacious, a different drug was prescribed. Six oral NSAIDs, previously reported to be efficacious in migraine and suggested by the guidelines, were prescribed: aspirin 500 mg, diclofenac 75 mg, ketorolac 20 mg, ibuprofen 600 mg, indomethacin 50 mg, naproxen 550 mg. Patients used the indicated drug for three consecutive migraine attacks. They were given a diary in order to record the clinical response in the three consecutive migraine attacks. According to current guidelines [27], a positive response was defined by having a decrease of ≥2 points in a 4-point scale intensity of pain or being pain-free at 2 h, after 120 min NSAID administration, in at least two attacks. The headache intensity scale was the ordinal 4-point scale, according to the current guidelines of the International Headache Society [27]: 0 =  no headache, 1 =  mild headache, 2 =  moderate headache and 3 =  severe headache. Partial response was defined as headache relief, defined as a reduction in the intensity of headache by 1 point, i.e., from moderate or severe at baseline to mild or none at 2 h after treatment.

Migraineurs were also prescribed one of the six oral triptans available in Italy (almotriptan 12.5 mg, eletriptan 40 mg, frovatriptan 2.5 mg, rizatriptan 10 mg, sumatriptan 50 mg, zolmitriptan 2.5 mg) for the other three consecutive migraine attacks (Figure S1 showed timeline and design of the study). Migraineurs were allowed to use a rescue medication, if the NSAID or triptan was inefficacious. Patients were instructed to use NSAIDs and triptans at the onset of the headache attack and not to consume any other rescue medication before 2 h after the first drug’s consumption.

Patients did not use any prophylactic medication and, whenever necessary, the prophylactic treatment was introduced after the pharmacological tests. Over the years, patients followed up at the Headache Center, with a number of visits, depending on the severity of their migraine. However, all patients were newly recalled at 6 months for a re-evaluation of their clinical conditions. Detailed headache characteristics were recorded using a standardized questionnaire. The clinical features of migraine are summarized in Table 1. Beck’s Depression Inventory (BDI) and the State-Trait Anxiety Inventory Form X (STAI-X)-1 and STAI-X2 were also administered.

### 4.2. Genotyping

Genomic DNA was extracted from 200 μL of peripheral EDTA blood using the QIAamp DNA Mini Kit (Qiagen S.p.A., Milan, Italy). After extraction, DNA samples were aliquoted and stored at −20 °C until use.

Primers were generated using the Primer3 software. Polymerase chain reactions (PCRs) were performed in a final volume of 25 mL, with 90 ng of genomic DNA, 0.2 unit of Taq Gold DNA polymerase, 250 nM of each primer, 1.5 mM MgCl_2_ and 50 mM dNTPs, 1X PCR buffer, and sequenced on an ABI3100 Genetic Analyzer (Applied Biosystems, Waltham, MA, USA).

PCR conditions were performed with initial denaturation at 95 °C for 10 min, 35 cycles at 95 °C for 1 min at a specific temperature for each primer, 72 °C for 1 min and a final elongation at 72 °C for 5 min. PCR products were electrophoresed on a 2% agarose 1× TBE gel and stained with ethidium bromide. Each patient was genotyped for six functional polymorphisms in the following genes: *IL-1α* (−889 C > T, rs1800587), *IL-1β* (−511 C > T, rs16944, and +3953 C > T, rs1143634), *IL-1RN* (VNTR), *IL-6* (−174 G > C, r1800795) and *TNF-α* (−308G > A, rs1800629), selected from the SNP database of the National Center for Biotechnology Information (NCBI; http://www.ncbi.nlm.nih.gov/ (accessed on 21 November 2022)) All polymorphisms were analyzed by enzymatic digestion. Some of the DNA samples were previously genotyped and reported in previous studies [20,28,29].

### 4.3. Statistics

Statistical analysis was performed using the Statistical Package for Social Sciences—version 26 (IBM SPSS Statistics 26, SPSS Inc., Chicago, IL, USA). Categorical variables were described as count and percentage. Continuous variables were described as mean and standard deviation or median and range. Differences in continuous data between groups were evaluated by the unpaired t-test or its non-parametric variant with the Mann–Whitney test. Furthermore, the chi-square test was used for differences in categorical variables. The Hardy–Weinberg equilibrium was verified for observed genotype frequency in all tested populations to detect deviation from the expected genotype distribution and to detect genotyping errors. The level of statistical significance was taken at *p* < 0.05.

## 5. Conclusions

To the best of our knowledge, this is the first study that has examined the influence of proinflammatory cytokine genes on the clinical response to NSAIDs in migraine attack. Our data support a role of functional polymorphisms in the *TNF-α* gene in the acute migraine attack. Further investigations are needed to better clarify the underlying neurobiological mechanisms of this effect.

## Figures and Tables

**Table 1:** Clinical characteristics and response to acute treatment. Data are displayed as mean ± standard deviation or count and percentage.

	Total (N = 313)	Male (N = 96; 30.7%)	Female (N = 217; 69.3%)	*p* Value
Age	38.36 ± 11.76	38.55 ± 12.05	38.30 ± 1.71	0.39
Age at onset	18.06 ± 9.61	17.74 ± 8.43	18.15 ± 9.94	0.22
N of annual migraine episodes	58.65 ± 37.69	58.27 ± 62.35	59.55 ± 4.19	0.09
Monthly migraine days (MMD)	4.89 ± 3.14	4.86 ± 5.20	4.96 ± 0.35	0.43
Headache duration (hours)	32.14 ± 27.10	26.21 ± 22.17	33.83 ± 28.17	0.009
Headache severe intensity	147 (47.0%)	40 (41.7%)	107 (49.3%)	-
Headache moderate intensity	156 (49.8%)	42 (43.8%)	114 (52.5%)	0.97
Headache mild intensity	165 (52.7%)	43 (44.8%)	122 (56.2%)	-
Unilateral pain	229 (73.2%)	75 (78.1%)	154 (70.8%)	0.21
Pulsating pain	225 (71.9%)	75 (78.1%)	150 (69.3%)	0.13
Photophobia	242 (77.3%)	68 (71.1%)	174 (80.2%)	0.07
Phonophobia	227 (72.5%)	66 (68.7%)	161 (74.4%)	0.34
Nausea	243 (77.6%)	65 (67.5%)	178 (82.1%)	0.007
Vomiting	159 (50.8%)	37 (38.4%)	122 (56%)	0.004
Smokers	67 (21.4 %)	21 (21.9 %)	44 (20.2%)	0.74
Non-responders to NSAIDs	65 (28.8%)	12 (12.5%)	53 (24.4%)	-
Responders to NSAIDs	123 (29.3%)	37 (38.5%)	86 (39.6%)	0.02
Partial responders to NSAIDs	125 (39.3%)	47 (49.0%)	78 (35.9%)	-
	Total (N = 290)	Male (N = 83; 28.6%)	Female (N = 207; 71.4%)	
Non-responders to triptans	80 (27.6%)	18 (21.7%)	62 (30.0%)	0.003
Responders to triptans	166 (57.2%)	43 (51.8%)	123 (59.4%)	
Partial responders to triptans	44 (15.2%)	22 (26.5%)	22 (10.6%)	

**Table 2:** Genotype frequencies (GF) and allele frequency (AF) of polymorphisms of *TNF-α* (−308 G > A, rs 1800629), *IL-1α* (−889 C > T, rs 1800587), *IL-1β* (−511 C > T, rs 16944 and +3953 C > T, rs 1143634), *IL-6* (−174 G > C, rs 1800795), in non-responders, responders and partial responders to NSAIDs. Data are displayed as count and percentage.

	GF			AF	
***TNF-α* (−308 G > A, rs1800629)**	G/G (%)	A/G (%)	A/A (%)	G	A
Non-responders	49 (75.4)	15 (23.1)	1 (1.5)	113 (87.6)	16 (12.4)
Responders	108 (87.8)	15 (12.2)	0 (0)	231 (93.9)	15 (6.1)
Partial responders	112 (89.6)	13 (10.4)	0 (0)	237 (94.8)	13 (5.2)
***IL-1α* (−889 C > T, rs1800587)**	C/C (%)	C/T (%)	T/T (%)	C	T
Non-responders	38 (58.5)	21 (32.3)	6 (9.2)	97 (74.6)	33 (25.4)
Responders	60 (48.8)	48 (39.0)	15 (12.2)	165 (67.0)	78 (33.0)
Partial responders	66 (52.8)	53 (42.4)	6 (4.8)	185 (74.0)	65 (26.0)
***IL-1β* (−511 C > T, rs16944)**	C/C (%)	C/T (%)	T/T (%)	C	T
Non-responders	36 (55.4)	22 (33.8)	7 (10.8)	94 (72.3)	36 (27.7)
Responders	57 (46.3)	51 (41.7)	15 (12.0)	165 (67.1)	81 (32.9)
Partial responders	64 (51.2)	57 (45.6)	4 (3.2)	185 (74.0)	65 (26.0)
***IL-1β* ( +3953 C > T, rs1143634)**	C/C (%)	C/T (%)	T/T (%)	C	T
Non-responders	41 (63.1)	23 (35.4)	1 (1.5)	105 (80.8)	25 (19.2)
Responders	68 (55.3)	38 (30.9)	17 (13.8)	174 (70.7)	72 (29.3)
Partial responders	70 (56.0)	45 (36.0)	10 (8.0)	185 (74)	65 (26)
***IL-6* (−174 G > C, rs1800795)**	C/C (%)	C/G (%)	G/G (%)	C	G
Non-responders	1 (1.5)	28 (43.1)	36 (55.4)	30 (23.1)	100 (76.9)
Responders	10 (8.1)	59 (48.0)	54 (43.9)	79 (32.1)	167 (67.9)
Partial responders	13 (10.4)	50 (40.0)	62 (49.6)	76 (30.4)	174 (69.6)

**Table 3:** Genotype frequencies (GF) and allele frequency (AF) of polymorphisms of *TNF-α* (−308 G > A, rs1800629), *IL-1α* (−889 C > T, rs1800587), *IL-1β* (−511 C > T, rs16944 and +3953 C > T, rs1143634), *IL-6* (−174 G > C, rs1800795), in non-responders, responders and partial responders to triptans. Data are displayed as count and percentage.

	GF			AF	
***TNF-α* (−308 G > A, rs1800629)**	G/G (%)	A/G (%)	A/A (%)	G	A
Non-responders	72 (90)	8 (10)	0 (0)	152 (95)	8 (5)
Responders	139 (83.7)	26 (15.7)	1 (0.6)	304 (91.6)	28 (8.4)
Partial responders	40 (10.1)	4 (9.1)	0 (0)	84 (95.5)	4 (4.6)
***IL-1α* (−889 C > T, rs1800587)**	C/C (%)	C/T (%)	T/T (%)	C	T
Non-responders	37 (46.3)	33 (41.3)	10 (12.5)	107 (66.9)	53 (33.1)
Responders	89 (53.6)	64 (38.6)	13 (7.8)	242 (72.9)	90 (27.1)
Partial responders	26 (59.1)	16 (36.4)	2 (4.5)	68 (77.3)	20 (22.7)
***IL-1β* (−511 C > T, rs16944)**	C/C (%)	C/T (%)	T/T (%)	C	T
Non-responders	41 (51.3)	31 (38.8)	8 (10.0)	113 (70.6)	47 (29.4)
Responders	81 (48.8)	71 (42.8)	14 (8.4)	233 (70.2)	99 (29.8)
Partial responders	24 (54.5)	18 (40.9)	2 (4.5)	66 (75.0)	22 (25.0)
***IL-1β* ( +3953 C > T, rs1143634)**	C/C (%)	C/T (%)	T/T (%)	C	T
Non-responders	46 (57.5)	24 (30.0)	10 (12.5)	116 (72.5)	44 (27.5)
Responders	94 (56.6)	60 (36.1)	12 (7.2)	248 (74.7)	84 (25.3)
Partial responders	28 (63.6)	13 (29.5)	3 (6.8)	69 (78.4)	19 (21.6)
***IL-6* (−174 G > C, rs1800795)**	C/C (%)	C/G (%)	G/G (%)	C	G
Non-responders	3 (3.8)	36 (45.0)	41 (51.3)	42 (26.3)	118 (73.8)
Responders	14 (9.3)	74 (44.6)	77 (46.4)	104 (31.3)	228 (68.7)
Partial responders	3 (6.8)	17 (38.6)	24 (54.5)	23 (26.1)	65 (73.9)

## Data Availability

Not applicable.

## Supplementary Materials

The following supporting information can be downloaded at: https://www.mdpi.com/article/10.3390/ijms24010657/s1.
