# Supplementary material for: Polymorphisms of the Proinflammatory Cytokine Genes Modulate the Response to NSAIDs but Not to Triptans in Migraine Attacks
Source: Int J Mol Sci. 2022 Dec 30;24(1):657. doi: 10.3390/ijms24010657 (PMC9820603; doi:10.3390/ijms24010657)

Supplementary Materials

Supplementary Table S1. Employment status of patients with migraine. Data are displayed as number and percentage. [a] Chi-square test.

Employment	Total (N=313)	Male (N=96)	Female (N= 217)	P value[a]
**Paid**	155 (49.5%)	56 (58.4%)	99 (45.9%)	0.029
**Self**	23 (7.4%)	5 (5.2%)	18 (8.3%)	
**Unemployed**	16 (5.1%)	_	16 (7.5%)	
**Student**	28 (8.9%)	10 (10.4%)	18 (8.3%)	
**Retired**	39 (12.5%)	8 (8.3%)	31 (14.2%)	
**Other**	52 (16.6%)	17 (17.7%)	35 (15.8%)	

Supplementary Table S2. Education levels of patients with migraine. Data are displayed as number and percentage. [a] Chi-square test.

Education	Total (N=313)	Male (N=96)	Female (N=217)	P value[a]
**Primary**	31 (9.9%)	12 (12.5%)	19 (8.9%)	0.250
**Middle**	102 (32.6%)	29 (30.2%)	73 (33.5%)	
**High**	153 (48.9%)	43 (44.8%)	110 (50.5%)	
**University**	27 (8.6%)	12 (12.5%)	15 (7.1%)	

Supplementary Table S3. Psychometric tests of patients with migraine. Data are displayed as mean ± standard deviation. [a] Unpaired t-test.

	Male (N=96)	Female (N=217)	P value[a]
**Beck Depression Inventory**	8.17 ± 3.39	10.74 ± 8.34	0.031
**State Trait Anxiety Inventory X1**	39.21 ± 10.69	40.89 ± 11.27	0.311
**State Trait Anxiety Inventory X2**	42.95 ± 10.92	45.29 ± 10.92	0.146

Supplementary Table S4. Genotype frequencies (GF) of polymorphisms of *IL-1RN* (VNTR), in non-responders, responders and partial responders to NSAIDs and triptans. Data are displayed as count and percentage.

	GF					
***IL-1RN* (VNTR)**	**1/1 (%)**	**1/2 (%)**	**1/3 (%)**	**1/4 (%)**	**2/2 (%)**	**2/3 (%)**
Non-responders to NSAIDs	34 (52.3)	19 (29.2)	5 (7.7)	1 (1.5)	5 (7.7)	1 (1.5)
Responders to NSAIDs	65 (52.8)	38 (30.9)	3 (2.4)	3 (2.4)	13 (10.6)	1 (0.8)
Partial responders to NSAIDs	61 (48.8)	39 (31.2)	5 (4.0)	9 (7.2)	11 (8.8)	0 (0)
Non-responders to triptans	48 (60.0)	22 (27.5)	2 (2.5)	2 (2.5)	6 (7.5)	0 (0)

Responders to triptans	78 (47.0)	52 (31.3)	7 (4.2)	10 (6.0)	18 (10.8)	1 (0.6)
Partial responders to triptans	23 (52.3)	13 (29.5)	4 (9.1)	0 (0)	3 (6.8)	1 (2.3)

Supplementary Figure S1. Timeline and design of the study.

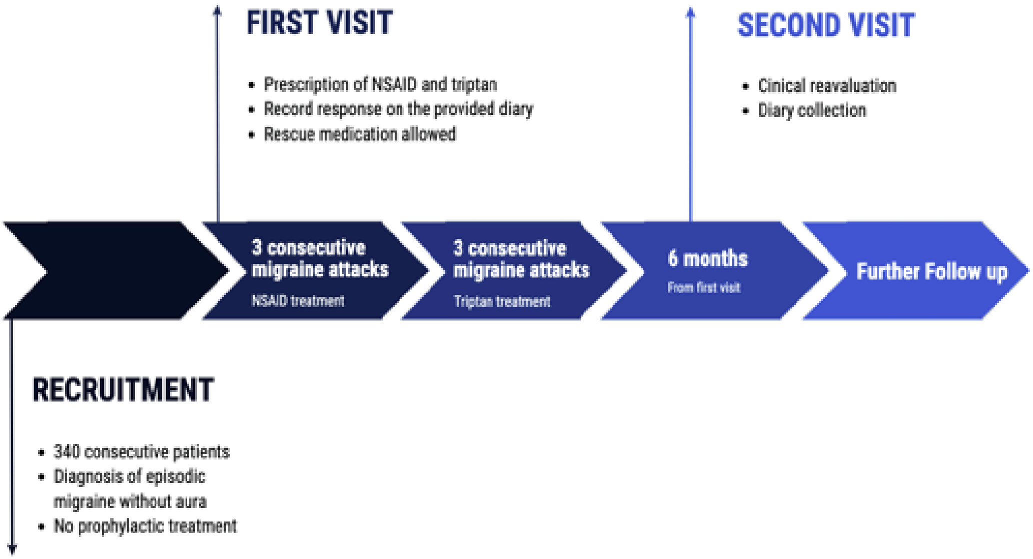

Supplement: Supplementary file 1 [file ijms-24-00657-s001.zip › ijms-2083132-supplementary.pdf]
